# Enrichment and comparative metagenomics of microbes involved in biocorrosion of gas transport or storage steel infrastructure

**DOI:** 10.3389/fmicb.2026.1771929

**Published:** 2026-03-10

**Authors:** Jackie Way, Taylor Sherman, Scott Leleika, Karen Crippen, Rebekah Wilson, Tekle T. Fida

**Affiliations:** 1 Environmental and Chemical Research Group, GTI Energy, Des Plaines, IL, United States; 2 U.S. Army Corps of Engineers, Champaign, IL, United States

**Keywords:** biofilm, culture enrichment, hydrogen sulfide, metagenome, microbiologically influenced corrosion

## Abstract

Biocorrosion, also known as microbiologically influenced corrosion (MIC), is the deterioration of metals caused by microbial activities that compromise the structural integrity, reliability, and safety of steel infrastructure. To identify the genetic determinants that MIC-causing microorganisms may use to attack steel infrastructure, field samples from natural gas infrastructure with a potential history of MIC were collected, enriched for different MIC categories, and subjected to whole-genome shotgun sequencing for metagenomic analysis. Biofilms were grown on carbon steel coupons or glass slides as attachment substrates to assess differences in microbial community composition and metabolic activities. The highest corrosion activities were observed in enrichments dominated by acid-producing bacteria (APB) and hydrogen-utilizing bacteria. APB enrichments resulted in the highest accumulation of organic acids and a severe decrease in culture fluid pH. A total of 57 metagenome-assembled genomes were recovered from the biofilms, some of which differed between carbon steel coupons and glass slide substrates. The metagenomes contained most of the known genes implicated in MIC and sulfide production, with substantial variation in estimated gene copy numbers among metagenomes and attachment substrates. Overall, comparative analysis of these biofilm metagenomes enriched from natural gas production and processing infrastructure highlights similarities to microbial communities commonly observed in oil production and processing systems and provides an overview of candidate genes that may be used as molecular probes for MIC.

## Introduction

Microbiologically influenced corrosion (MIC), also known as biocorrosion, is defined as the deterioration of metal due to microbial activity, and it is a result of the interactions between the metal surface, abiotic corrosion products, bacterial cells, and their metabolites (Beech and Sunner, 2004). Corrosion due to MIC is estimated at about 20%–30% of the $2.9 trillion estimated global cost of corrosion (Koch et al., 2016; Little et al., 2020; Omar et al., 2021). MIC can occur in environments where no other types of corrosion are observed, and in combination with other types of abiotic corrosion. MIC is known to induce a localized attack and can accelerate the kinetics of anodic/cathodic corrosion reactions. MIC activities begin with the attachment of planktonic cells to steel infrastructure, where microbes can grow, reproduce, consume nutrients, and produce biofilm (Khouzani et al., 2019). Bacterial biofilms influence corrosion by altering the chemistry around the metal surface, and the extracellular polymeric matrix of biofilm is known to bind metals via phosphate, sulfate, and carboxyl groups (Avidan et al., 2010). The accumulation of extracellular polymeric substances can chelate metal ions and lead to increased corrosion (Dong et al., 2011). Higher rates of corrosion of carbon steel have been observed with mixed bacterial cultures compared to pure cultures, demonstrating the complexity of the MIC process and how the microbes can interact synergistically with each other to act on substrates (Beech and Sunner, 2004). Although carbon steel is the focus of many studies, microbes can also contribute to the corrosion of other metals such as aluminum, copper, nickel alloys, and titanium. The economic and environmental impacts of MIC span across many sectors such as oil and gas infrastructure, water utilities, power plants, offshore wind turbines, and even medical and dental devices (Xu et al., 2023).

While information on abiotic corrosion and management strategies has improved over the past several decades, MIC remains a complex and high-priority issue. There is a pressing need to expand research into the underlying mechanisms of MIC in order to develop better detection and mitigation strategies. Oxidation of metallic iron with sulfate as an electron acceptor is regarded as the principal reaction in anaerobic corrosion induced by sulfate reducing bacteria (SRB) (Rabus et al., 2013). SRB undergo dissimilatory sulfate reduction by coupling iron oxidation to sulfate reduction and producing sulfide (also known as souring) as a metabolite byproduct. In addition to souring, the produced sulfide reacts with metals that causes corrosion with precipitation of metallic iron as FeS (Kreitman et al., 2016). Although most studies of MIC focused on the corrosive effects of SRB, MIC also involves other groups of microorganisms such as sulfur oxidizing bacteria (SOB), acid producing bacteria (APB), iron reducing bacteria (IRB), iron oxidizing bacteria (IOB), thiosulfate reducing bacteria (TRB), nitrate reducing bacteria (NRB), methanogenic archaea, acetogens, fungi, microalgae, and others (Telegdi et al., 2020). In addition to the corrosive effects of sulfide by SRB, the proposed MIC process involves the microbial production of metabolites that enhance the oxidation to ferrous iron with the reduction of H^+^ to H_2_, H_2_-mediated electron transfer between the metal and microbes, direct metal-to microbe electron transfer, and redox-active organic molecules shuttling electrons between the metal and the microbes (Enning and Garrelfs, 2014; Mand and Enning, 2021; Mand et al., 2014; Xu et al., 2023).

Many studies have been focused on the MIC activities of SRB from oil production or processing facilities. Information is limited for the MIC activities of the other types of MIC-causing microbes, especially for gas production and processing facilities. In this study, field samples from gas infrastructure that showed a potential history of biocorrosion were obtained and enriched for MIC activities by different groups of MIC-causing microbes to better understand the MIC process and how different groups of microbial communities and populations act to initiate and propagate metallic biocorrosion. A subset of the samples was inoculated into culture media with conditions that favored the growth of specific group of MIC-causing microbes in the presence and absence of carbon steel material. Subsequent enrichments and transfers were made to promote the selective growth of specific microbes in the presence of either glass slides (as a control) or carbon steel coupons. After a specific period of incubation, the biofilms were scraped from the slides and coupons, and subject to whole genome shotgun sequencing and metagenome analysis, with the intention of elucidating how microbial populations differed in the presence of metal, and any genes that could be involved in MIC.

## Materials and methods

### Sample collection and 16S rRNA sequencing

A total of 32 solid and liquid pipeline and gas storage infrastructure samples were previously collected from the interiors or exteriors of visually corroded gas transporting pipelines or storage infrastructure across selected states in the United States. The samples included soils within immediate proximity to the corroded pipelines, liquid from gas storage and processing facilities, pipeline sludges, and scrapes from failed gas transmission pipelines. Samples were transported to the laboratory on ice within 24 h and stored at 4 °C until processing. These samples were collected to determine the presence of MIC-associated microbes. Gene abundances of MIC-associated microbes in these 32 samples were previously reported (Fida et al., 2024). Of these, five samples were selected based on the 16S rRNA gene copy numbers and used for MIC enrichment and metagenomic analysis. Depending on sample quantities obtained from the field, genomic DNA was isolated from 1–100 mL of each liquid, or 0.1–0.5 g of solid samples using the FastDNA extraction kit for soil (MP Biomedicals), according to the manufacturer’s instructions. DNA was quantified with a Qubit fluorimeter (Invitrogen) using the Quant-iT double-stranded DNA (dsDNA) HS assay kit (Invitrogen). The DNA was shipped to Omega Bioservices for sequencing and microbial community analysis. Sequencing was performed by targeting the V3–V4 region of 16S rRNA genes using the MiSeq (PE300) platform. The 16S rRNA genes were amplified through two-step PCR reactions sequentially with nonbarcoded and barcoded primers according to an Omega Bioservices proprietary procedure. Quality control, sequence identification, and bioinformatic analysis were performed by Omega Biosciences using Illumina BaseSpace applications. Microbial community abundances were expressed as percentage abundances of the reads belonging to each taxon within an individual sample.

### Microbial enrichments

MIC enrichments were performed using Coleville synthetic brine medium K (CSBK) (Callbeck et al., 2013) containing different electron donors and acceptors or carbon sources to meet the specific growth requirements of selected MIC-causing microbes. The media (per liter) contained 1.5 g NaCl, 0.05 g KH_2_PO_4_, 0.32 g NH_4_Cl, 0.21 g CaCl_2_·2H_2_O, 0.54 g MgCl_2_·5H_2_O, and 0.1 g KCl. After autoclaving, sterile 1 M NaHCO_3_ (30 mL) and 1 mL of trace mineral supplements (ATCC, Manassas, VA) were added and the pH was adjusted to 7.6 using HCl solution. Samples for the enrichment process were selected based on 16S rRNA gene copy numbers and microbial community abundances. Five different sample that showed at least 1 × 10^5^ 16S rRNA gene copies per mL or had high relative abundance of candidate microbes were chosen for the enrichment of different types of MIC-causing microbes (Table 1). The enrichments include SRB, APB, hydrogen utilizing bacteria (HUB), and methanogens (MET). In the scope of this study, HUB encompasses a broad category of microbes that can metabolize hydrogen as an energy source.

**Table 1:** Samples selected for enrichment of different types of MIC-causing microbes.

Sample name	Sample source (State)	Sample type	Month and year collected	Total bacteria (16S rRNA) gene copies per mL	Enrichment used
Debris.C.07	Virginia	Soil in the vicinity of corroded pipeline	November 2022	1.62E+10	SRB1
Liquid.C.02	Virginia	Sludge from gas transporting pipeline	December 2022	8.10E+08	SRB2 and MET
Liquid.C.04	Florida	Liquid from corroded pipeline	December 2022	1.03E+08	HUB1
Debris.C.02	Iowa	Debris from corroded pipeline	April 2022	1.67E+07	HUB2
CrudeFail.01	Louisiana	Corrosion debris from failed crude transporting pipeline	March 2023	5.86E+06	APB

The microbes were enriched in 160 mL capacity serum bottles containing 110 mL of growth media. To enrich SRB, sodium sulfate (5 mM) was used as an electron acceptor and volatile fatty acids (VFA: 5 mM each of acetate, butyrate, and propionate) were used as electron donors. To enrich HUB, sodium sulfate (5 mM), a carbon steel coupon, and headspace CO_2_ (20%) were used as the electron acceptor, electron donor, and carbon source, respectively. Yeast extract (1 g/L) and dextrose (10 g/L) were used to enrich methanogens and APB, respectively.

Biofilms were allowed to grow on carbon steel coupons (C1018) blasted with fine glass beads (Metal Samples Company, AL) for each enrichment media inoculated with their respective samples in an anaerobic chamber. The coupons were cleaned by sonication in isopropyl alcohol for 2 min, air dried, weighed in triplicate to determine the average starting weight, and then fully immersed in the media. All electron donors, electron acceptors, and carbon sources were added through syringes into the CSBK media. The serum bottles were flushed with a gas mix containing 90% N_2_ and 10% CO_2_ (N_2_–CO_2_ gas mix) to create an anaerobic environment, capped with butyl rubber stoppers, and sealed with an aluminum crimp sealer. The absence of oxygen was confirmed with an oxygen sensor in a COY anaerobic chamber, and redox indicators were not added to the media. The serum bottle headspace for HUB enrichment was replaced with 20% CO_2_. The cultures were incubated in the dark at 30 °C for 3 weeks, after which the coupons were removed and processed. The coupon surfaces were scraped using sterile swabs to remove the biofilm-forming cells on the coupons and resuspended in 10 mL of fresh CSBK media by vortexing. A 2 mL volume of the resuspended biofilm cells from each enrichment was used to inoculate fresh media to continue the subsequent enrichments with a new coupon. After 4 subsequent transfers, the final enrichment cultures (2 mL of the biofilm cells) were transferred to large anaerobically sealed bottles (500 mL capacity) containing 350 mL of CSBK to compare growth and activity in the presence and absence of carbon steel coupons. Electron donors and acceptors for each MIC type were kept the same, apart from the SRB and HUB, which had 10 mM of sulfate and 10 mM of VFA added for the final enrichment. For each type of microbe, a pair of carbon steel coupons or glass slides were placed into enrichment bottles. Two controls, one with N_2_–CO_2_ gas mix and one with additional 20% headspace CO_2_, without inoculum sources were included to determine abiotic corrosion activities. Each enrichment on carbon steel coupons and glass slides was conducted in biological duplicates. For HUB grown in the absence of carbon steel coupons (grown on glass slides), a gas mix containing hydrogen (60%) and CO_2_ (40%) was added into the headspace. The headspaces of the remaining bottles were flushed with a N_2_–CO_2_ gas mix.

After 3 weeks of incubation, the coupons and glass slides were removed, and the biofilm cells were harvested as described above. For each enrichment, the biofilm was harvested from one of the pairs of coupons or slides and used for DNA extraction and metagenomic analysis. The other pair of coupons were used for scanning electron microscopy (SEM) imaging. To determine the corrosion rate, the coupons used for DNA extraction were wiped down to remove any residual debris and moisture and processed according to procedures established elsewhere (Fida et al., 2021). Briefly, the coupons were sonicated in a 15% HCl solution containing 5 g/L of corrosion inhibitor (1,3-di-n-butyl-2 thiourea) for 2 min, sonicated in 1 M sodium bicarbonate for 2 min, rinsed with deionized water, and sonicated in isopropyl alcohol for 2 min. The cleaned coupons were then allowed to dry under air stream, weighed in triplicate, and averaged to determine the final weights and corrosion rates. The corrosion rate (mm/y) was calculated using the ASTM standard weight loss method, as given by CR = (K × W)/(A × T × D), where W is the weight loss (g), A is the exposed surface area (cm^2^), T is the exposure time (h), D is the material density (g/cm^3^), and K is a unit conversion constant (87,600).

### Organic acids, pH, and sulfide assays

Samples (1 mL) were withdrawn from duplicate enrichment cultures at the end of the experiments and used to determine sulfide concentration and production of organic acid ions, such as acetate, butyrate, propionate, and lactate. The aliquot samples were filtered using 0.2-μm nylon membrane filters (Fisher Scientific, NH) to remove cells and debris. The organic acid concentrations were determined from the filtered samples (300 μL) using a high-performance liquid chromatography (HPLC) system equipped with a Vanquish Diode Array Detector at 210 nm and an Acclaim™ Organic Acid (250 × 4.0 mm) column (Thermo Fisher) with a flow rate of 1.0 mL/min. The samples were acidified using 20 μL of 1 M H_3_PO_4_ prior to analysis. Mobile phases were 25 mM KH_2_PO_4_ (pH 2.5) and acetonitrile. The pH of the culture fluid was determined immediately at the end of the experiment using a Metrohm 914 pH/Conductometer (Metrohm) according to the manufacturer’s protocol. Sulfide concentrations were determined colorimetrically using diamine reagents as described elsewhere (Fida et al., 2018). Briefly, 20 μL of sample was added to 200 μL of 0.1 M zinc acetate to capture the total dissolved sulfide. Then, 200 μL of diamine reagent (2 g/L N, N-dimethyl-*p*-phenylenediamine in 20% H_2_SO_4_) and 10 μL of iron alum solution (0.2 M NH_4_Fe(SO_4_)_2_. 12H_2_O in 2% H_2_SO_4_) were added to form methylene blue color. The intensity of the resulting blue coloration developed after 10 min of incubation at room temperature was measured spectrophotometrically at an absorbance of 670 nm.

### Scanning electron microscopy

The carbon steel coupons containing the biofilm cells were subjected to SEM (Hitachi S-3500 N) to image the coupons and observe the physical nature of the biofilms. After removal from the enrichment culture, the coupons were stored overnight in a 2.5% glutaraldehyde solution, which was used as a fixative agent for microbial biofilm. The following day, the coupons were dehydrated using an ethanol dilution series, where the coupons were exposed to 50% ethanol for 5 min, 70% ethanol for 5 min, 95% ethanol for 10 min, and 100% ethanol for 10 min. The coupons were sputter coated in gold using the PELCO SC-7 sputter coater according to the manufacturer’s protocol, after which SEM imaging and elemental mapping was performed.

### DNA extraction and processing

DNA was extracted from duplicates of the biofilms grown on carbon steel coupons and glass slides. The coupons or glass slides were carefully removed from the bottles and immersed into 0.1 M phosphate-buffered saline solution. Biofilm cells were then scraped off using sterile cotton-tipped wood applicators followed by brief vortexing, then each set of duplicates were pooled together prior to sequencing. Genomic DNA was isolated using the FastDNA extraction kit for soil (MP Biomedicals) according to the manufacturer’s instructions. The concentrations of DNA were quantified with a Qubit fluorimeter using the Quant-iT double-stranded DNA (dsDNA) HS assay kit (Invitrogen). The DNA samples were shipped to Omega Bioservices for metagenomic sequencing.

### Metagenome analysis

Whole genome shotgun sequencing of the DNA extracted from the biofilm cells was performed by Omega Bioservices, who provided the raw FASTQ files with whole genome DNA sequencing reads and a MultiQC report (Ewels et al., 2016). ATLAS (v.2.18.1), a software package that provides a complete workflow for metagenome data processing, was used to obtain quality controlled reads from the raw FASTQ files, assemble reads into contigs, bin the metagenome-assembled genomes (MAGs), and perform functional annotations (Kieser et al., 2020). The ATLAS pipeline was run with the default parameters and settings in the generated config files. Given the number of samples run in parallel, SPAdes (v.3.15.5) (Bankevich et al., 2012) was used for assembly, and vamb (Nissen et al., 2021) was used for binning.

The assembled sequences and post-filter coverage statistics files produced for each microbial enrichment sample by ATLAS were submitted to the Integrated Microbial Genomes and Metagenomes (IMG) system (Chen et al., 2023). Metadata for the metagenome samples were submitted to the Genomes Online Database (GOLD) and IMG, validated, and annotated by the IMG Annotation Pipeline (v.5.2.1). ATLAS files were uploaded to KBase (Arkin et al., 2018) for analysis. CheckM (v1.0.18) was used to assess the quality of the genome bins produced by ATLAS (Parks et al., 2015). GTDB-Tk (v1.7.0) was used to obtain taxonomic assignments of the bacterial and archaeal genomes based on the Genome Taxonomy Database (GTDB) version R06-RS202 (Chaumeil et al., 2022). Scripts from ATLAS were run to produce relative abundance graphs for microbial community analysis of each sample. The raw FASTQ files with whole genome DNA sequencing reads were deposited in the Sequence Read Archive (SRA) at NCBI with accession number PRJNA1346464 or via direct link of https://www.ncbi.nlm.nih.gov/sra/?term=PRJNA1346464.

## Results and discussion

### Sample selection and microbial community analysis

**Figure 1:**
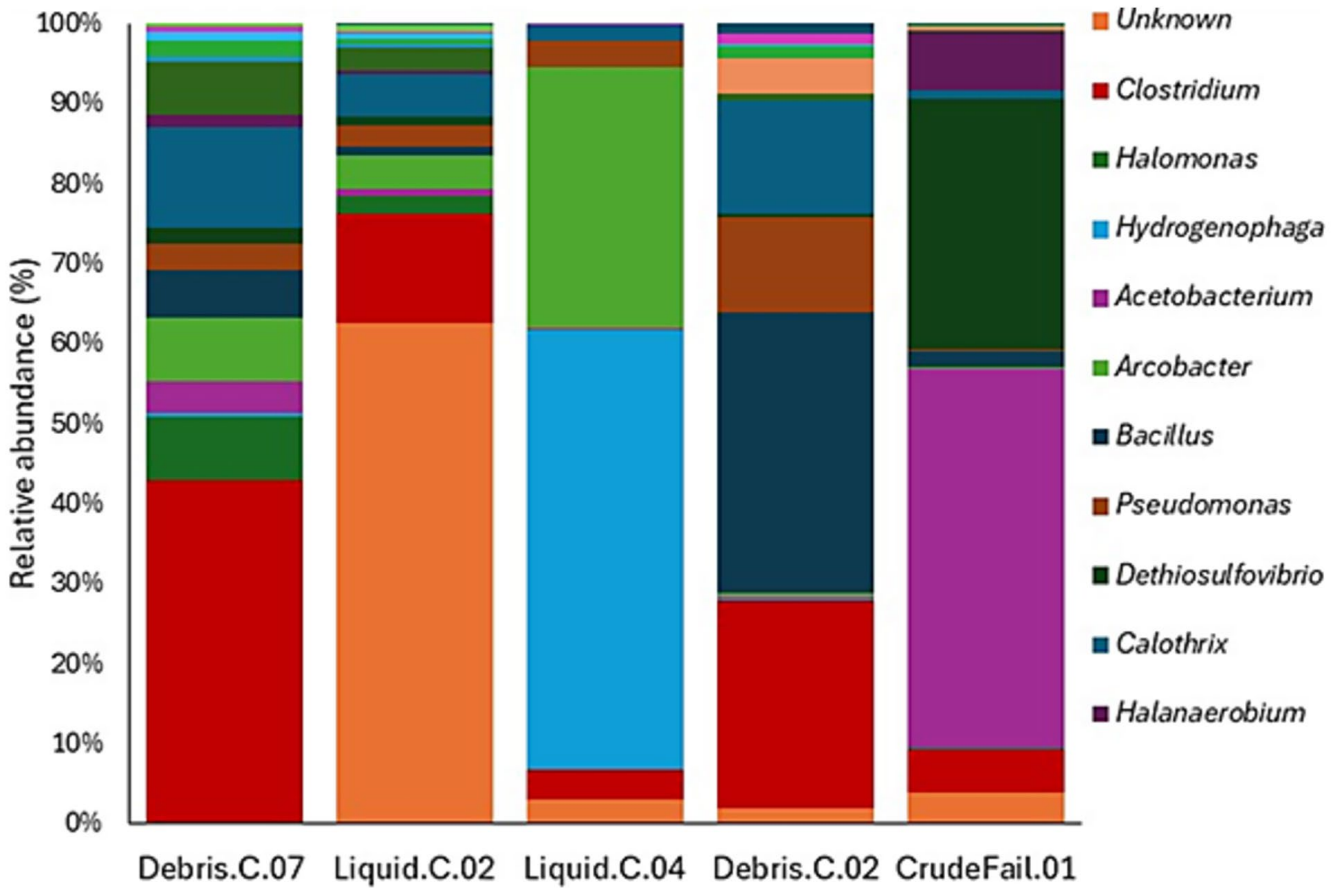
Microbial community composition of samples before enrichment. Microbial community composition of samples obtained from potentially biocorroded natural gas processing and transporting facilities and selected for MIC enrichment. Microbial taxa are presented at the genus level for known taxa and designated as unknown for unassigned taxa name.

Samples for MIC enrichment were obtained from solid or liquid contents of corroded gas transporting pipelines, gas storage facilities, or surrounding soil that showed a potential history of MIC activities. Microbial community analysis of the samples was conducted before selecting them for the enrichment processes. The community composition (Figure 1) differs among the different sample types (solids and liquids) and sources. Uncharacterized microbial taxa constitute up to 63% in some of the samples such as Liquid.C.02. This sample also contains up to 14% of *Clostridium*. Debris.C.07 constitutes the most diverse group of microorganisms, *Clostridium* being the most dominant taxa. Liquid.C.04 is mainly dominated by two groups of microbes, *Hydrogenophaga* (55%) and *Arcobacter* (33%). *Clostridium*, *Bacillus*, *Pseudomonas*, and *Calothrix* are the most common taxa observed in Debris.C.02. CrudeFail.01 is also dominated by two groups of microbes, *Acetobacterium* (47%) and *Dethiosulfovibrio* (31%). *Hydrogenophaga* has been identified as a known genera of hydrogen-oxidizing bacteria and plays crucial roles in biogeochemical cycling of H_2_ and CO_2_ in various environments (Li et al., 2025). *Clostridium*, *Acetobacterium*, and *Pseudomonas* are also known for acid production and are the major concern for biocorrosion (Gottwald and Gottschalk, 1985; Maji and Lavanya, 2024; Ramos Monroy et al., 2019; Wang et al., 2023). Unlike most microbial communities observed in oil production and processing facilities (Santos et al., 2020; Tian et al., 2017), the dominance of SRB was not observed in the samples before enrichment, except *Dethiosulfovibrio*. Based on the observed microbial abundance data, a total of 6 MIC enrichment conditions were set up using 5 samples. These include two for SRB (Debris.C.07 and Liquid.C.02), two for HUB (Liquid.C.04 and Debris.C.02), one for APB (CrudeFail.01), and one for methanogens (Liquid.C.02).

### Enrichment of MIC-associated microbes

**Figure 2:**
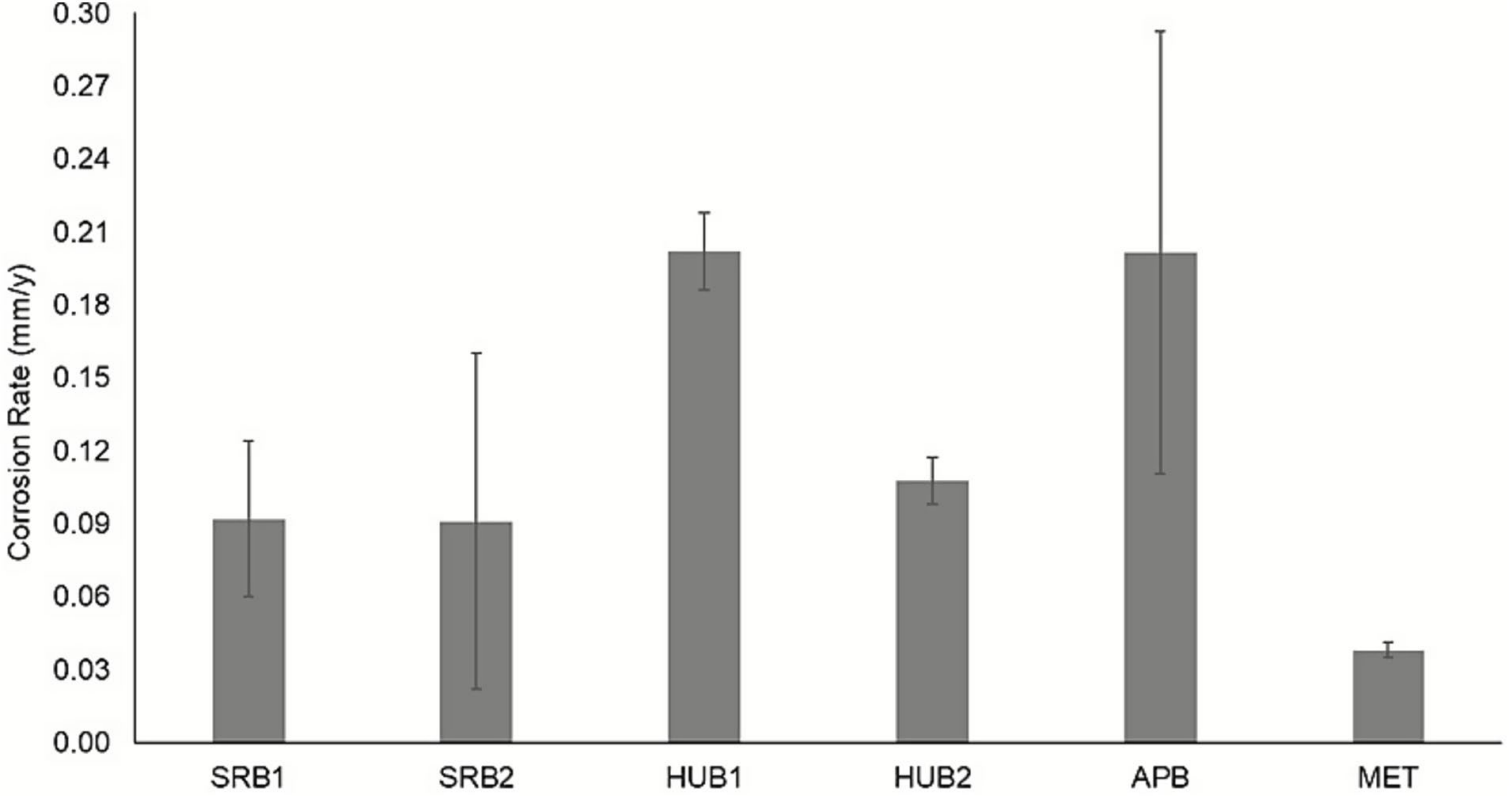
Corrosion rate of coupons. Carbon steel coupon corrosion rate after the final subsequent transfer of the different microbial enrichments cultures. Error bars represent the range between duplicate samples.

Growth of SRB and HUB enrichments were visually monitored from the formation of iron sulfide as a black precipitate on the coupon or in the culture media, whereas MET and APB were monitored based on turbidity of the culture media for subsequent transfers. Cleaned carbon steel coupons indicated some etching of the metal surfaces after exposure to the enrichment cultures. The etching was particularly prominent for APB, SRB, and HUB coupons (Supplementary Figure S1). Subsequent enrichments showed concomitant increases in corrosion rate for HUB. This is possibly due to the competitive advantage of HUB to use electrons directly from the metal surface in the absence of organic carbon sources as an electron donor for sulfate reduction. The highest corrosion rates (Figure 2) on the final enrichment process were recorded for APB (0.201 ± 0.091 mm/y) and HUB1 (0.202 ± 0.016 mm/y), whereas the lowest corrosion rate was observed for MET (0.0380 ± 0.003 mm/y) (Figure 2). The corrosion rates are also different depending on the sample source for the same type of MIC-causing microbe enrichment. Due to the inherently selective nature of the enrichment process, the results represent potential MIC-driving consortia rather than conclusive results about the original field structures. Abiotic controls in the presence of an additional 20% CO_2_ in the headspace showed a higher corrosion rate than the N_2_–CO_2_ headspace, indicating the corrosive effect of CO_2_ on the carbon steel coupons.

### Change in pH of culture media

**Figure 3:**
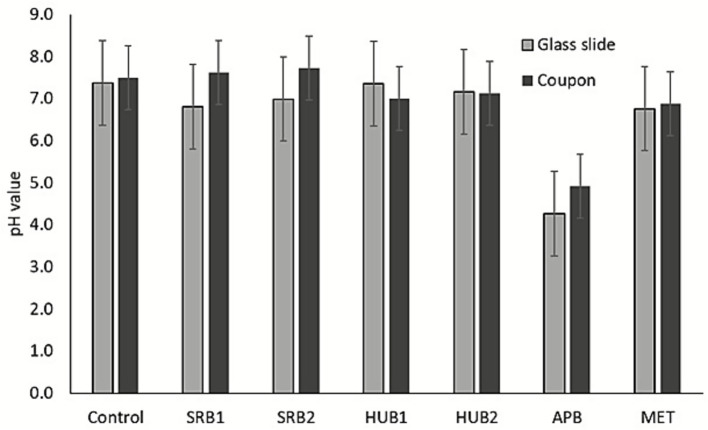
Change in pH of culture media. pH value of culture media after enrichment of the different MIC-causing microbes in the presence of carbon steel coupons and glass slides. Error bars represent the range between duplicate samples.

Differences in pH value of the culture media were observed at the end of the enrichments of the different MIC-causing microbes (Figure 3). A slight increase in pH was observed for most of the enrichments in the presence of carbon steel coupons compared to glass slides, except for HUB where the pH slightly decreased for one of the samples. APB severely decreased the pH of the culture media from about 7.5 to 4.25 and 4.91 in the presence of glass slides and coupons, respectively. The decrease in pH of the culture media corroborates the increase in corrosion rates induced by APB activities. In addition to the biofilms formed by APB, the acidic conditions created during microbial growth could promote corrosion of metals by destabilizing the protective films or coatings of the metal surface and modification of localized environment at the metal/solution interface (Khouzani et al., 2019; Madirisha et al., 2022).

### Hydrogen sulfide (H_2_S) production

**Figure 4:**
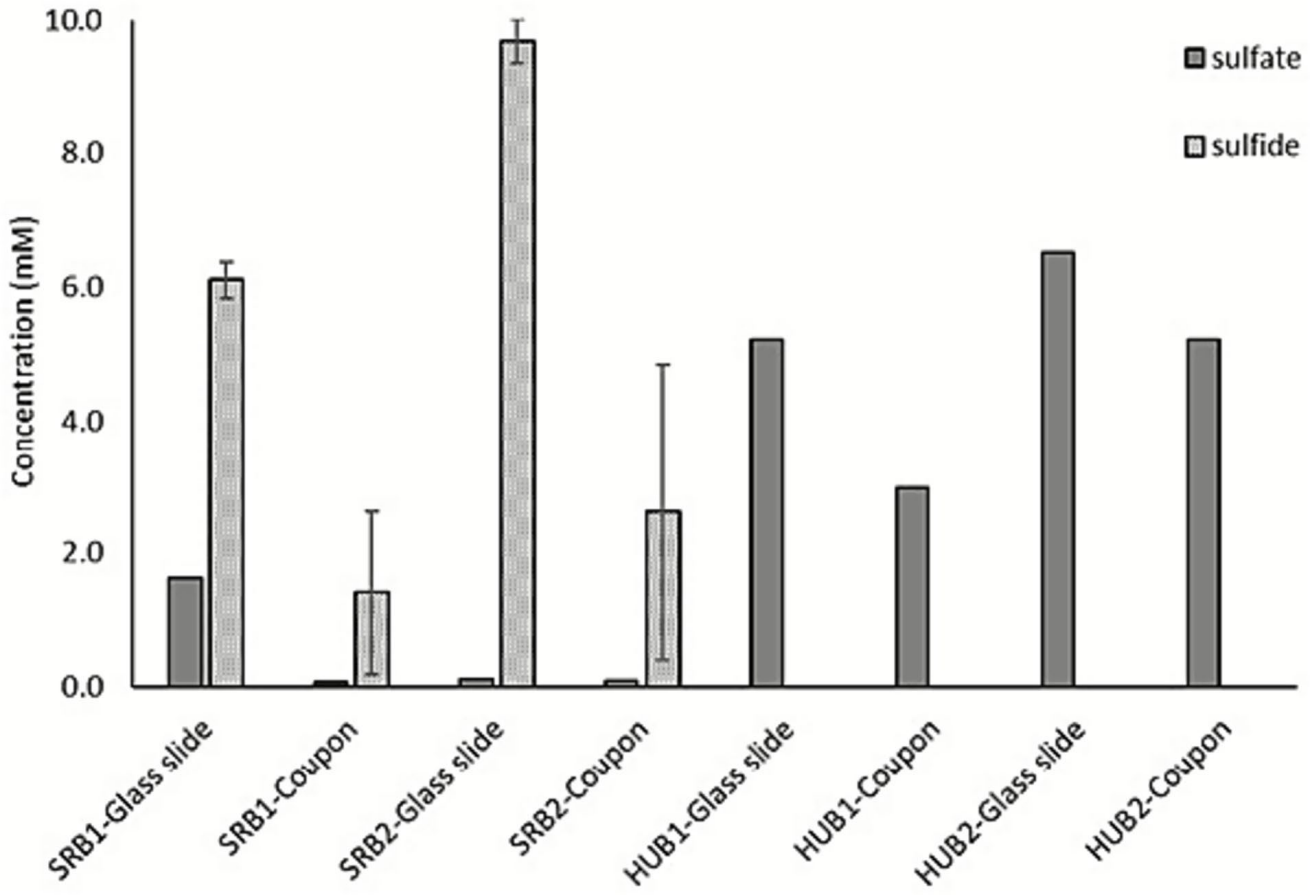
Sulfate and sulfide concentrations. Sulfate and aqueous H_2_S detected in SRB and HUB enrichment cultures in the presence of carbon steel coupons and glass slides. Error bars represent the range between duplicate samples.

Aqueous sulfide concentration was determined for the enrichments to which sulfate (10 mM) and VFA (10 mM) were added. Up to 10 mM H_2_S was detected for the SRB enrichment at the end of incubation for 3 weeks in sealed bottle bioreactors in the absence of carbon steel coupons (on glass slides) indicating stoichiometric reduction of sulfate (Figure 4). However, a maximum of 2.6 mM of H_2_S was detected for the SRB enrichment in the presence of carbon steel coupons suggesting that majority of the produced H_2_S reacted with iron and formed FeS. This is also evident from the formation of the black precipitate in the solution (Supplementary Figure S2). H_2_S was not detected in HUB enrichment solutions although up to 60% of the sulfate was reduced. More sulfate reduction was observed in the presence of coupons compared to the glass slides for both the HUB enrichment. The sulfide produced in HUB enrichment might have precipitated as FeS or was oxidized in the presence of hydrogen.

### Organic acid analysis

**Figure 5:**
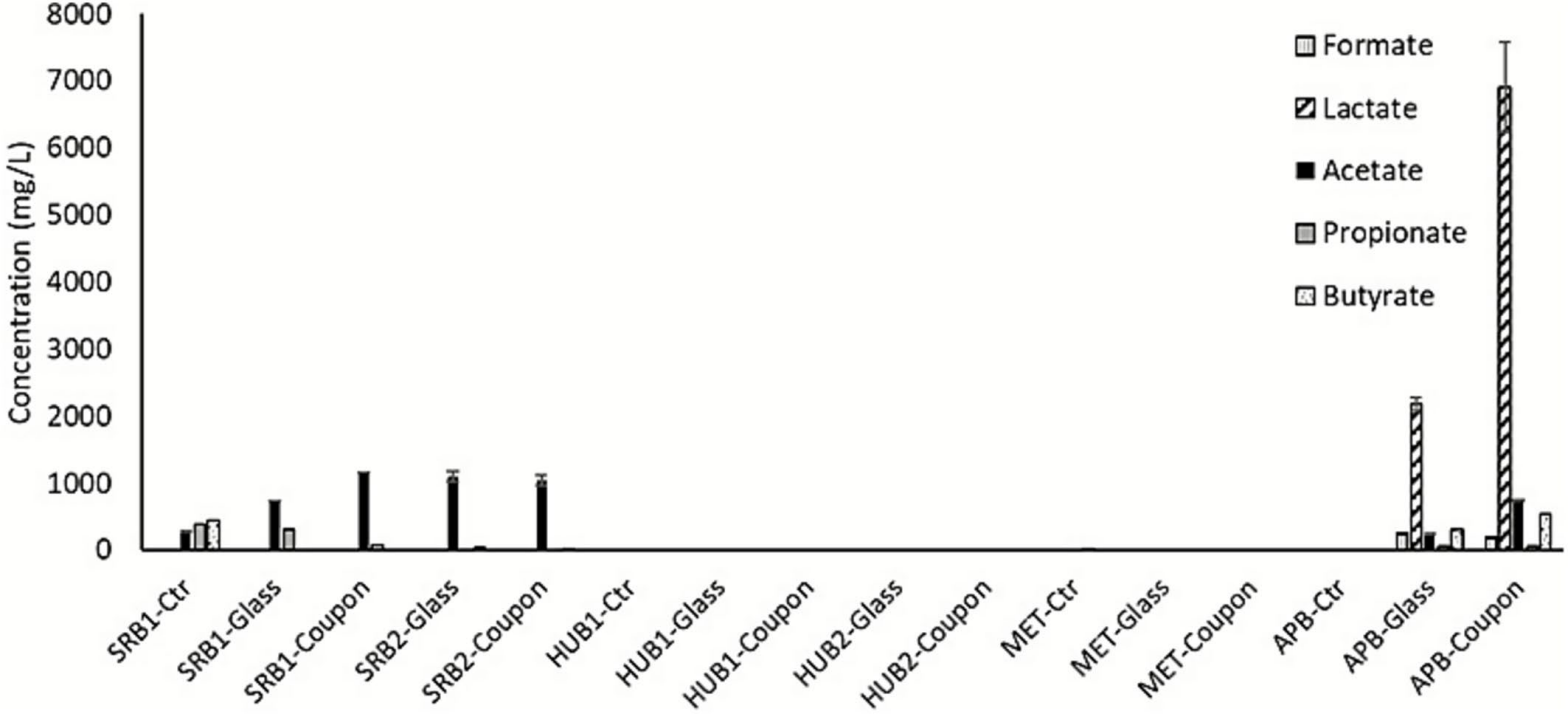
Organic acid production. Accumulation of organic acids in the culture media after enrichment of MIC-associated microbes in the presence of carbon steel coupons and glass slides. Error bars represent the range between duplicate samples.

Acetate was observed as a metabolite product of VFA oxidation by both SRB enrichment cultures. Organic acid production was not observed during the growth of HUB and MET enrichments. VFAs were not provided as carbon sources for these two cultures, although yeast extract (1 g/L) was provided for MET. Surprisingly, APB produced significantly high amounts of lactate (up to 7 g/L) during growth on dextrose (10 g/L) (Figure 5). Lactate production was about 3-fold higher during growth on carbon steel coupon than glass slides. Production of other organic acids, such as formate, acetate, butyrate, and propionate were also observed during growth of APB on dextrose, although the concentrations were less than 0.5 g/L. The accumulation of organic acids corroborates the decrease in pH and the increase in corrosion rates induced by APB activities. These findings suggest that APB induce higher corrosion than the other types of MIC-causing microorganisms in the presence of adequate carbon sources. This is mainly due to the production of organic acids as corrosive metabolites (Khouzani et al., 2019; Madirisha et al., 2022).

### SEM imaging of biofilms and elemental mapping

**Figure 6:**
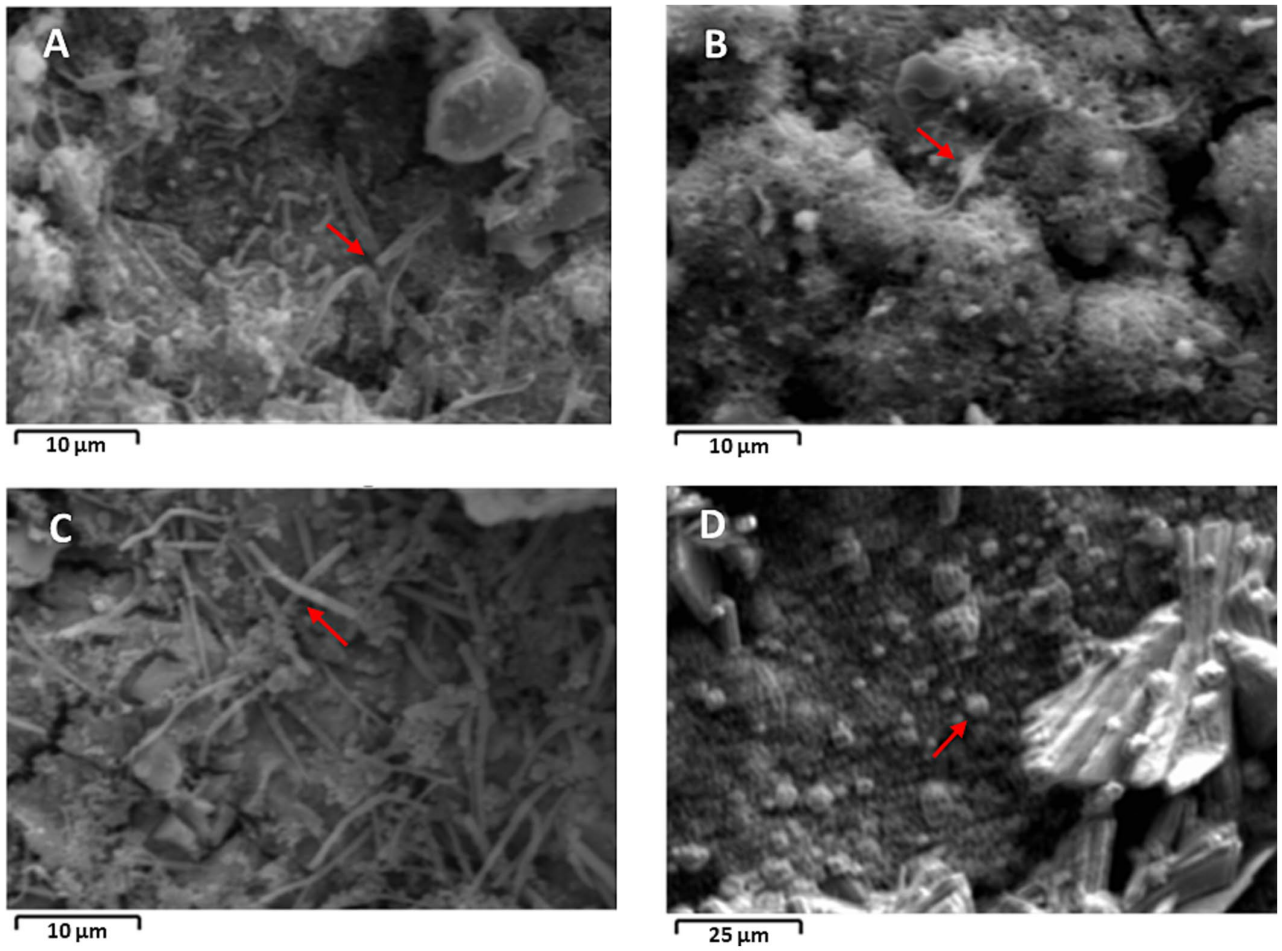
SEM images of biofilm grown on carbon steel coupons. SEM image of: **(A)** SRB, **(B)** HUB, **(C)** APB, and **(D)** MET. Red arrows indicate visible bacteria with flagellar/pili networking.

Images of selected coupons from each MIC enrichment culture were taken using SEM to provide visual observations of the biofilms formed on the coupons. Images of biofilms grown on glass slides were not captured because of the reflection of beams (conductivity issues), making it difficult to obtain quality images. The different MIC enrichment types showed differences in biofilm or cell morphologies, such as bacilli (rod) and cocci (spherical) shapes (Figure 6). Visual observation of the growth of microbes on the metal surface and their communication through flagellar/pili networking as an electron transfer mechanism is also visible from the images (Figures 6A–C). Microbes within the biofilm communicate through secretion of chemical signaling molecules that convey specific messages important for the formation, development, functional maintenance, and structural integrity of the biofilm (Moons et al., 2009; Qi et al., 2025; Summers et al., 2010). Many studies reported the importance of flagellar or pili networking for electron transfer or shuttle between microorganisms, within individual cells, and between microbes and metal surfaces (Holmes et al., 2022; Malvankar et al., 2011; Qi et al., 2025).

Elemental mapping of the biofilms grown on carbon steel coupons showed the presence of elements, such as Fe, C, O, S, and P (Supplementary Figure S3). As expected, the most abundant element detected across the enrichment samples was Fe, the highest concentration being detected in HUB enrichment. The presence of high iron concentrations in the biofilm is also in agreement with the high corrosion rate observed as indicated above.

### Metagenome analysis

#### Metagenome analysis of APB

**Figure 7:**
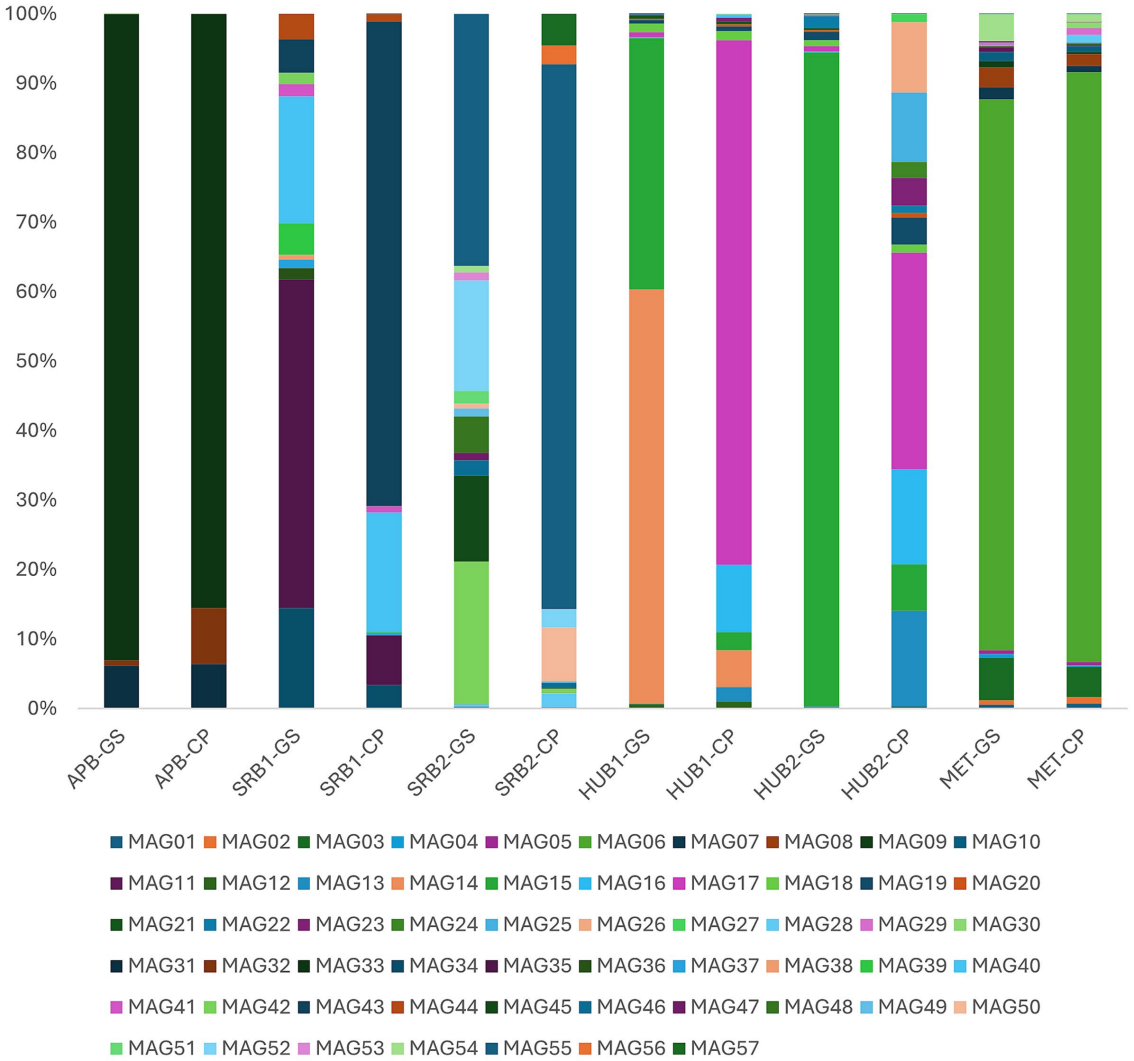
Relative abundances of the MAGs in the biofilm grown on carbon steel coupons and glass slides for each enrichment culture of APB, SRB, HUB, and MET. Details of the taxonomy of each MAG with its enrichment source are shown in Supplementary Table S1.

Taxonomic analysis of the DNA extracted from the APB enrichment culture biofilm (APB-GS representing APB biofilm grown on glass slides and APB-CP representing APB biofilm grown on carbon steel coupons) identified a total of 8 different MAGs (APB-GS contained 8 MAGs, and APB-CP contained 6 MAGs), with 6 of the MAGs observed in both APB-GS and APB-CP (Figure 7). Three of the MAGs (two belonging to the genus *Clostridium*, the other being *Sporolactobacillus nakayamae*) represent over 99.9% of the enrichment culture population (Figure 7). These microbes are known to produce organic acids implicated in MIC (Li et al., 2024; Ramos Monroy et al., 2019). *S. nakayamae* was the most prominent microbe, making up 93% of the metagenome for APB-GS and 85.5% of the metagenome for APB-CP.

IMG’s functional annotations for APB-GS and APB-CP yielded 13,918 and 13,612 protein coding genes, respectively. Genes encoding acetate kinase (*ackA*), butyrate kinase (*buk*), and lactate dehydrogenases were found abundantly in the APB samples regardless of attachment substrate. These genes are essential for organic acid metabolism and are used as molecular probes for APB monitoring (Davis et al., 2024; Fida et al., 2024; Zhu, 2007, 2008). An abundance profile search in IMG estimated the gene copies (how many times certain genes were present in the metagenome), normalizing the number of copies based on the genome size and read depth. APB-GS had an estimated 4,263 gene copies of *ackA*, 318 gene copies of *buk*, 4,656 gene copies of L-lactate dehydrogenase, and 6,404 copies of D-lactate dehydrogenase. In contrast, APB-CP had an estimated 3,253 gene copies of *ackA*, 239 gene copies of *buk*, 3,431 gene copies of L-lactate dehydrogenase, and 4,484 copies of D-lactate dehydrogenase. The abundance of organic acid metabolism genes in APB enrichment corroborates the increased pH of the culture media regardless of the attachment substrates.

**Figure 8:**
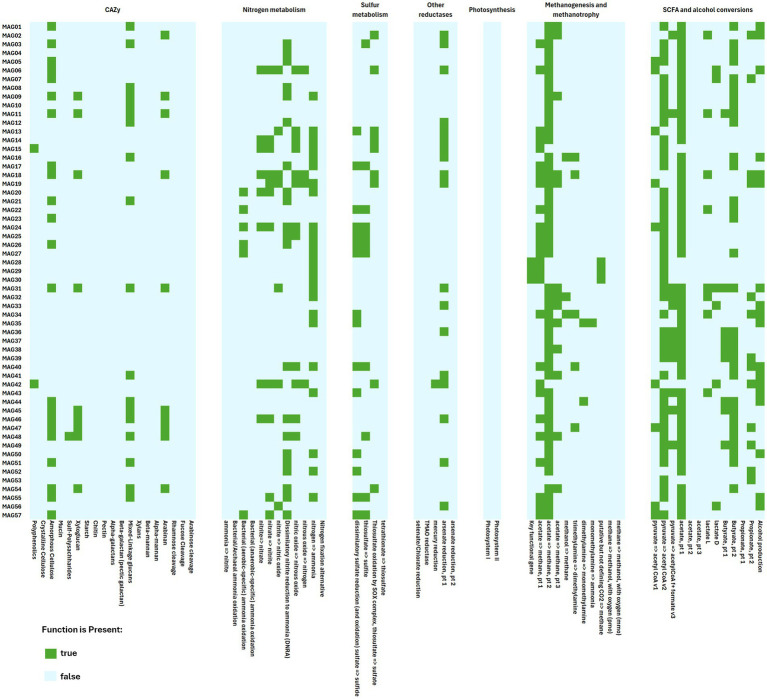
Functional annotation of MAG. An overview of functional annotations with KEGG and CAZy to infer pathways and KEGG modules for each generated MAG, highlighting each MAG’s metabolism. The sulfur metabolism and SCFA and alcohol conversion KEGG modules highlight activity involving sulfide, sulfate, and organic acids.

Both APB enrichments have high estimated copies of genes that fall into the Kyoto Encyclopedia of Genes and Genomes (KEGG) (Kanehisa et al., 2024) pathways for membrane transport, such as ABC transporters, the phosphotransferase system (PTS), and the bacterial secretion system (Figure 8). ABC transporters are a family of bacterial efflux pumps, which are important for cation exchanges and metal metabolism. Genes relating to the ABC-type multidrug transport system and secretion have been observed in microbial consortia from the seabed of an oil drilling platform, and have been hypothesized to contribute to biofilm formation and ion exchange (Song et al., 2021). The PTS system is known to regulate the biofilm formation, catalyze the transport and phosphorylation of sugar derivatives, and regulate processes such as quorum sensing molecules, and the metabolism of carbon, nitrogen, and phosphorus (Dang and Lovell, 2016). Some of these gene products observed in the APB enrichments include ATP-binding cassette (subfamily B, multidrug efflux pump), oligopeptide transport system substrate-binding protein, energy-coupling factor transport system permease protein, and L-cystine transport system permease protein. Gene products for the PTS include fructose PTS system EIIBC or EIIC component, mannose PTS system EIID component, mannose PTS system EIIC component, mannose PTS system EIIAB component, fructose PTS system EIIA component, and galactitol PTS system EIIC component. Both APB enrichments also have high estimated gene copies for type IV secretion system protein VirD4, and genes involved in the KEGG pathways for quorum sensing and biofilm formation. Quorum sensing signaling molecules are important in biofilm formation, as they are secreted by bacterial cells and contribute to reduced flagellar mobility and increased extracellular polymeric substance (EPS) secretion which has been known to enhance MIC (Scarascia et al., 2019).

#### Metagenome analysis of SRB

DNA extracted from the biofilms of both SRB enrichments (SRB1, SRB2) were dominated by sulfur disproportionating bacteria, regardless of the attachment substrate. In total, 34 different MAGs were found among the four different SRB enrichment. These include 15 for SRB1-GS, 15 for SRB1-CP, 21 for SRB2-GS, and 19 for SRB2-CP. In both SRB populations, the cultures grown on carbon steel coupons had a less diverse population of microbes compared to the cultures grown on glass slides (Figure 7). This difference suggests that the growth on coupons tends to enhance the selection of biofilms from specific microbial taxa that are implicated in sulfate reduction, or biocorrosion of steel. This is in agreement with a previous report indicating that SRB grown in the presence of carbon steel showed decreased diversity of microbial community with the dominance of certain SRB species after corrosion (Hua et al., 2024). The dominant MAG in SRB1-GS belonged to the genus *Desulfofarcimen*, constituting 47.3% of the population. The presence of *Desulfofarcimen* dropped to 7.2% in SRB1-CP, and the dominant MAG for SRB1-CP was from the family *Desulfotomaculales*, making up 69.8% of the population. The genus *Desulfosporosinus* was found at similar levels in both SRB1 enrichments (18.3% in SRB1-GS and 17.1% in SRB1-CP). The genus *Desulfobulbus* constituted 36.2% of the SRB2-GS population, and increased to 78.5% in SRB2-CP. All these microbes are known genera of SRB and are implicated in MIC and souring (Barton and Fauque, 2022).

The SRB1 enrichment yielded 55,255 protein coding genes for SRB1-GS and 38,940 protein coding genes for SRB1-CP whereas the SRB2 enrichment yielded 61,438 protein coding genes for SRB2-GS and 34,307 protein coding genes for SRB2-CP. An abundance profile search showed that SRB1-CP and SRB2-CP had higher numbers of estimated gene copies for dissimilatory sulfate reduction, such as adenylylsulfate reductase subunits A and B (*aprA*, *aprB*), sulfate adenylyltransferase (*sat*, *met3*), and dissimilatory sulfite reductase alpha and beta subunits (*dsrA*, *dsrB*). The enrichments on glass slides (SRB1-GS and SRB2-GS) contained genes mapping to the sulfur-oxidation (SOX) system which were not detected for enrichment on carbon steel coupons. This suggests that some sulfur oxidation may be initiated by SRB when carbon steel is absent to serve as an additional energy or electron source for sulfate reduction. Genes encoding hydrogenase (NAD^+^, ferredoxin), cytochrome-*c3* hydrogenase, ferredoxin hydrogenase, coenzyme F420 hydrogenase, sulfhydrogenase, and proton-translocating NAD(P)^+^ transhydrogenase were found in the SRB enrichments. Sulfhydrogenase was found at higher levels in SRB1-CP compared to SRB1-GS, but the gene copies did not differ significantly between the SRB2 biofilms. The hydrogenases are utilized to catalyze hydrogen acquisition from the metal surface by many of the MIC-causing microbes, such as SRB and MET (Tsurumaru et al., 2018; Xu et al., 2023).

A total of 472 different enzymes were found to have higher estimated gene copies in both SRB1-CP and SRB2-CP compared to their glass slide counterparts. A KEGG pathway search in IMG indicated that those involved in biofilm formation included: adenylate cyclase, histidine kinase, starch synthase, glycogen phosphorylase, hydrolases (acting on carbon-nitrogen bonds, other than peptide bonds), anthranilate synthase, non-specific serine/threonine protein kinase, transferases/glycosyltransferases/hexosyltransferases, protein-serine/threonine phosphatase, serine-O-acetyltransferase, phenylacetate-CoA ligase, and protein-secreting ATPase (Figure 8). Genes encoding different categories of ABC transporter were also found in higher estimated copies in the SRB enrichments. Upregulations of ABC transporter genes have been reported for *D. vulgaris* during growth on carbon steel and have been implicated in the biogenesis of cytochromes to facilitate electron transport system (Scarascia et al., 2019).

#### Metagenome analysis of HUB

A total of 24 MAGs were observed across the HUB enrichments. These include 19 for HUB1-GS, 17 for HUB1-CP, 24 for HUB2-GS, and 20 for HUB2-CP. The biofilm on both HUB enrichments (HUB1, HUB2) grown on glass slides was dominated by the genus *Sulfuricurvum*, constituting over 94% of both populations (Figure 7). However, the HUB enrichments grown on carbon steel coupons showed a large decrease in *Sulfuricurvum* (6%) in the microbial community. HUB1-CP was mainly dominated by the genus *Humidesulfovibrio* (75.6%) and *Acetobacterium* (9.6%). *Humidesulfovibrio* made up 31.2% of the HUB2-CP population, with *Sideroxydans* and *Acetobacterium* each constituting about 14%. *Humidesulfovibrio*, originally assigned as *Desulfovibrio*, is implicated in sulfate, thiosulfate, sulfite, and elemental sulfur reduction using *c*-type cytochromes (Sass et al., 2009). *Acetobacterium* have been observed to enhance iron corrosion (Philips et al., 2018) and *Sideroxydans* is commonly associated with iron oxidation (Procópio, 2019). Enrichments of HUB on glass slides were dominated by one or a few microbes whereas enrichment on carbon steel coupons showed more diverse taxa. The diverse taxa observed on carbon steel coupons could be due to the growth advantage of microbes with the potential of utilizing hydrogen from metal surfaces as an electron donor for sulfate reduction activities. *Sulfuricurvum* is a known sulfur oxidizing bacterium (Dang and Lovell, 2016; Fida et al., 2021; Kodama and Watanabe, 2004) and this might be the reason for absence of sulfide in the culture media.

**Figure 9:**
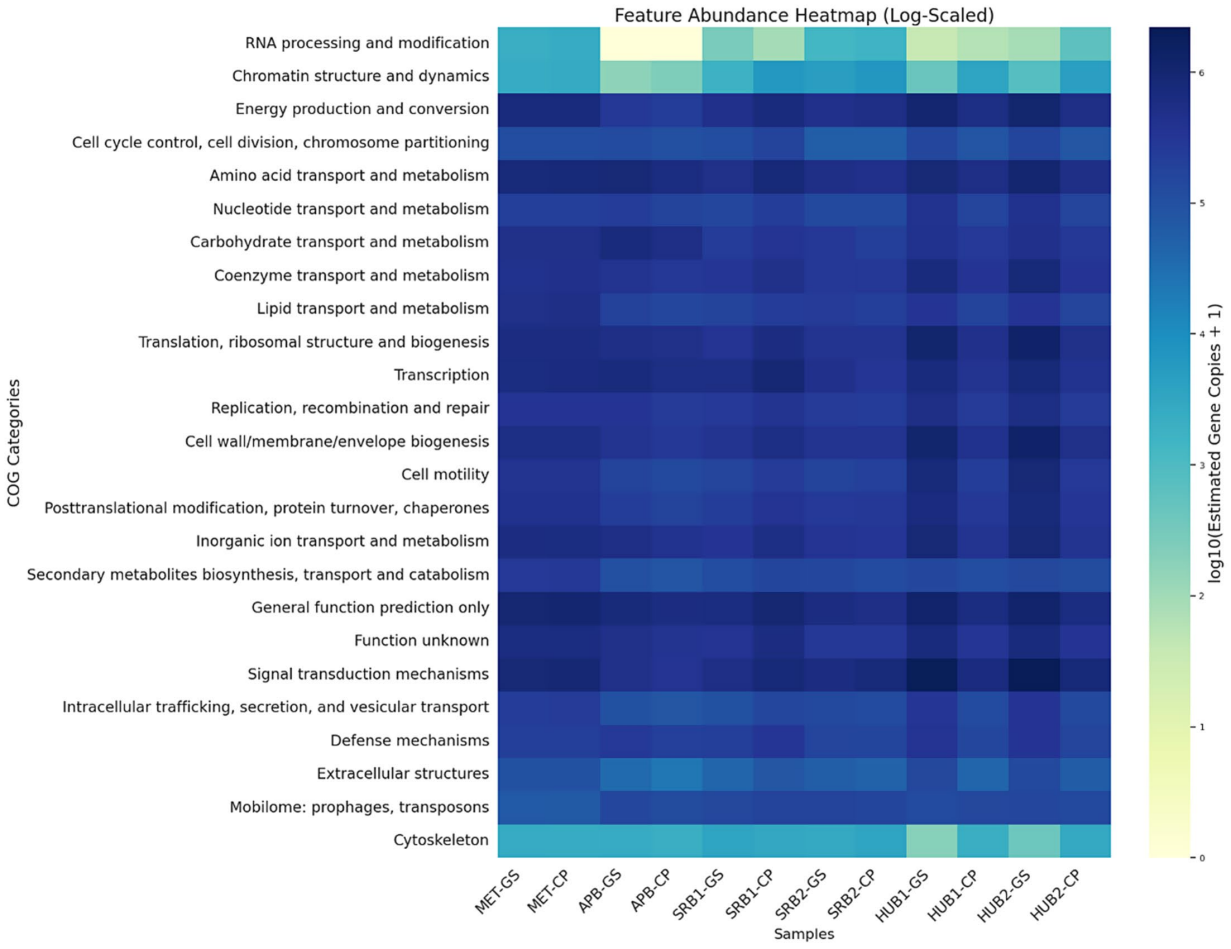
Heatmaps of genes by COG category. Heatmaps with estimated gene copies for all metagenome samples, sorted by COG category. The COG categories provide a functional overview and grouping method for the types of genes found in each enrichment.

The first HUB enrichment (HUB1) yielded 28,499 protein coding genes for HUB1-GS and 49,550 protein coding genes for HUB1-CP. The second HUB enrichment (HUB2) yielded 43,706 protein coding genes for HUB2-GS and 54,367 protein coding genes for HUB2-CP. Both HUB enrichments grown on carbon steel coupons had higher estimated gene copies for the KEGG modules related to dissimilatory sulfate reduction, acetyl-CoA pathway (CO_2_ to acetyl-CoA), and methanogenesis (CO_2_ to methane) compared to the HUB enrichments grown on glass slides. HUB1-CP and HUB2-CP were seen to have lower sulfate concentrations compared to their glass slide counterparts, suggesting higher rates of sulfate reduction. HUB1-CP, which had a high prominence of *Humidesulfovibrio*, had high measured corrosion rates. Genes encoding hydroxyacid-oxoacid transhydrogenase, cytochrome-*c3* hydrogenase, ferredoxin hydrogenase, coenzyme F420 hydrogenase, sulfhydrogenase, hydrogenase (acceptor), NAD(P)^+^ transhydrogenase (Si-specific), and proton-translocating NAD(P)^+^ transhydrogenase were found in the HUB enrichments (Figure 8). Cytochrome-*c3* hydrogenase, ferredoxin hydrogenase, and sulfhydrogenase were found to have much higher estimated gene copies in HUB1 and HUB2 grown on carbon steel coupons, compared to the growth on glass slides in the presence of CO_2_ and H_2_. These genes are implicated in electron transfer and the electrical MIC (EMIC) corrosion process. EMIC is a corrosion mechanism related to lithotrophic growth where SRB withdraw electrons directly from iron whereas chemical MIC (CMIC) is related the corrosion process due to production of corrosive metabolites such as H_2_S or organic acids (Enning and Garrelfs, 2014). HUB grown on glass slides have high estimated gene copies for the COG categories for signal transduction mechanisms, cell wall/membrane/envelope biogenesis, cell motility (Figure 9) compared to their carbon steel counterparts and all other microbial enrichment types, indicating an increased presence of genes involved in biofilm formation. HUB1-GS and HUB2-GS have higher estimated copies of genes involved in sulfur metabolism, methane metabolism, and carbon metabolism. Both HUB enrichments on glass slides have estimated copies of over 10 different dehydrogenases, including malate dehydrogenase, NADPH dehydrogenase (quinone), succinate dehydrogenase, and fumarate reductase. The increased abundance of carbon, methane and hydrogen metabolism during growth on glass slide is likely due to hydrogenotrophic methanogenesis process involved in the conversion of CO_2_ and H_2_ in the headspace into methane or organic acids (Šmigáň et al., 1990; Zabranska and Pokorna, 2018) although organic acids were not detected in the culture. The produced organic acids could serve as electron donors for sulfate reduction by SRB (Chen et al., 2017).

#### Metagenome analysis of MET

The biofilm that grew on the glass slides and carbon steel coupons in the methanogen enrichments yielded a diverse group of MAGs with MET-GS constituting 27 MAGs and MET-CP constituting 29 MAGs (Figure 7). The most prominent microbe in the methanogen enrichment was a *Pseudomonas* species, making up 79.2% of the MET-GS biofilm and 84.8% of the MET-CP biofilm. *Pseuodomonas*, such as *P. aeruginosa,* has been previously reported to produce biofilms on steel surfaces and accelerate corrosion (Xu et al., 2017). *Methanococcus vannielii* and microbes from the genera *Methanocalculus* and *Methanospirillum* were present in the enrichment, although they made up a small percentage of the microbial population (0.48% of the enrichment with glass slides, and 2.92% of the enrichment with carbon steel coupons). These methanogens are known to be members of microbial communities that grow on and corrode metallic surfaces (Mei et al., 2024).

The methanogen enrichments grown on glass slides yielded 58,988 protein coding genes while growth on carbon steel coupons resulted in 56,966 protein coding genes. An abundance profile search estimated 237 gene copies of methyl-coenzyme M reductase in MET-CP and 33 gene copies in MET-GS. In general, MET-CP had higher estimated gene copies of genes involved in methane metabolism compared to MET-GS (Figure 8). The increased presence of methanogens in the enrichment with carbon steel coupons suggests that the metal is important to their sustained growth. MET-CP had higher estimated gene copies of methyl-coenzyme M reductase and formylmethanofuran dehydrogenase compared to MET-GS, but acetyl-CoA synthetase gene copies were similar between the two groups, suggesting that in the presence of metal, the microbial community relied more on CO_2_ to produce methane. A BLASTP search was conducted in IMG to identify a multiheme *c*-type cytochrome (*mmcA*) implicated in direct electron uptake and transfer from the metal surface by many MIC-causing microbes (Qi et al., 2025). Hits were found in MET regardless of attachment substrate although identity percentages were low, ranging from 22% to 37%.

## Conclusion

The samples selected for microbial enrichments from gas production and transporting infrastructure identified a wide variety of MIC-causing microbes, many of which were not taxonomically characterized and represented up to 60% in some of the samples. *Clostridium*, *Hydrogenophaga*, *Acetobacterium*, and *Halomonas* were prominent in most of the samples. Throughout the subsequent enrichments, the highest corrosion rates were observed in APB and HUB enrichments. APB enrichment also resulted in a drastic decrease of culture fluid pH and the highest accumulation of organic acids, such as lactate. SEM images of the biofilms that grew on the carbon steel coupons showed the presence of thick biofilm with possible microbial networking structure for electron shuttling. Elemental mapping of the biofilms resulted in high levels of iron in the biofilms, indicative of active corrosion processes.

The metagenome analysis of the post-enrichment samples recovered a diverse range of microbes between each enrichment type, demonstrating that the enrichment conditions successfully resulted in the selective growth of different types of MIC-causing microbes. The presence or absence of metal as an iron or electron source for enrichment of MIC-causing microbes caused a shift in both the SRB and HUB populations. SRB populations became much less diverse when incubated in the presence of carbon steel coupons with the microbial community dominated by 1 or 2 specific MAGs. The reverse happened with the HUB population, where the microbial community became more diverse in the presence of carbon steel coupons suggesting coordinated microbial efforts to acquire electrons from the metal surface when there is no easily available electron source. Metagenome analysis of the different MIC microbes also indicated the presence and abundance of many of the known MIC genetic determinants. These include genes implicated in dissimilatory sulfate reduction for SRB, acetate kinase and butyrate kinase for APB, hydrogenases and *c*-type cytochromes for HUB, and multiheme *c*-type cytochrome for MET. Some of these genes, such as hydrogenases and *c*-type cytochromes were abundantly present in all MIC enrichment types implicating the possibility of using these genes as universal molecular probes for monitoring and control of MIC. These estimated gene copy numbers reflect genetic potential, and further studies into gene expression and activity are required to validate the role of highlighted genes of interest in active MIC mechanisms. Overall, these comprehensive MIC enrichment processes demonstrated how microbial communities could adapt to the different growth conditions in the presence and absence of metal. The metagenome analysis successfully inferred many candidate genetic determinants and pathways for future study, with electrochemical and expression-based validation being required to confirm the importance of the genes. Future research is also required to integrate this inferential metagenomic data with metatranscriptome analysis to identify and validate universal target genes for MIC monitoring and controls.

## Data Availability

The datasets presented in this study can be found in online repositories. The names of the repository/repositories and accession number(s) can be found in the article/Supplementary material.
